# Supplementary material for: Prevalence of sleep apnea in children and adolescents in Colombia according to the national health registry 2017–2021
Source: PLoS One. 2022 Aug 31;17(8):e0273324. doi: 10.1371/journal.pone.0273324 (PMC9432726; doi:10.1371/journal.pone.0273324)
Supplement: Attachment — Submitted filename: Response to reviewers.docx [file pone.0273324.s002.docx]

Dr. Kuo-Cherh Huang

Academic Editor

PLOS ONE

PONE-D-22-09943

Prevalence of sleep apnea in children and adolescents in Colombia according to the national health registry, 2017-2021

Dear Dr. Kuo-Cherh Huang

Academic Editor

PLOS ONE

We thank the reviewers and the journal’s editorial team for their valuable comments. Please find attached a revised version of the manuscript. Below you can find a point-by point response to the reviewers’ comments.

We look forward your response.

Kind regards,

The authors

Journal Requirements:

When submitting your revision, we need you to address these additional requirements.

1. Please ensure that your manuscript meets PLOS ONE's style requirements, including those for file naming. The PLOS ONE style templates can be found at

<https://journals.plos.org/plosone/s/file?id=wjVg/PLOSOne_formatting_sample_main_body.pdf> and

<https://journals.plos.org/plosone/s/file?id=ba62/PLOSOne_formatting_sample_title_authors_affiliations.pdf>

RESPONSE: Thank you for the observation. We have reviewed the PLOS ONE style requirements. We have made corrections in file naming and in the manuscript following the style templates.

2. Please provide additional details regarding participant consent. In the ethics statement in the Methods and online submission information, please ensure that you have specified (1) whether consent was informed and (2) what type you obtained (for instance, written or verbal, and if verbal, how it was documented and witnessed). If your study included minors, state whether you obtained consent from parents or guardians. If the need for consent was waived by the ethics committee, please include this information.

If you are reporting a retrospective study of medical records or archived samples, please ensure that you have discussed whether all data were fully anonymized before you accessed them and/or whether the IRB or ethics committee waived the requirement for informed consent. If patients provided informed written consent to have data from their medical records used in research, please include this information.

RESPONSE: We provided additional details regarding participant consent. We included a new Ethical considerations and Data availability statement sub heading in the revised manuscript as follows: The study protocol was reviewed and approved by the Research and Ethics Committee of Hospital Universitario San Ignacio and Pontificia Universidad Javeriana, both located in Bogota, Colombia. (FM-CIE-0473-21). The study was classified as no risk research and conducted in agreement with the Helsinki Declaration and Resolution 008430 of 1993 issued by the Colombian Ministry of Health. Data collected for analysis came from SISPRO, the Colombian national health registry [17]. Data is fully anonymized in the source, before being accessed by researchers. Thus, a waiver for informed consent was obtained. The raw data is available publicly or under request at <https://www.sispro.gov.co>

3. In your Data Availability statement, you have not specified where the minimal data set underlying the results described in your manuscript can be found. PLOS defines a study's minimal data set as the underlying data used to reach the conclusions drawn in the manuscript and any additional data required to replicate the reported study findings in their entirety. All PLOS journals require that the minimal data set be made fully available. For more information about our data policy, please see <http://journals.plos.org/plosone/s/data-availability>.

Upon re-submitting your revised manuscript, please upload your study’s minimal underlying data set as either Supporting Information files or to a stable, public repository and include the relevant URLs, DOIs, or accession numbers within your revised cover letter. For a list of acceptable repositories, please see <http://journals.plos.org/plosone/s/data-availability#loc-recommended-repositories>. Any potentially identifying patient information must be fully anonymized.

Important: If there are ethical or legal restrictions to sharing your data publicly, please explain these restrictions in detail. Please see our guidelines for more information on what we consider unacceptable restrictions to publicly sharing data: <http://journals.plos.org/plosone/s/data-availability#loc-unacceptable-data-access-restrictions>. Note that it is not acceptable for the authors to be the sole named individuals responsible for ensuring data access.

We will update your Data Availability statement to reflect the information you provide in your cover letter.

RESPONSE: Thank you for the comment. We have updated the Cover letter with a Data Availability Statement. Data collected for analysis came from SISPRO, the Colombian national health registry. Data in SISPRO is fully anonymized irreversibly before being accessed by researchers. The raw data is available publicly or under request at https://www.sispro.gov.co . We have attached a supplement with the data set underlying the results. We also included the SISPRO website within the manuscript in which interested researchers could gain access to the information.

4. Please include your full ethics statement in the ‘Methods’ section of your manuscript file. In your statement, please include the full name of the IRB or ethics committee who approved or waived your study, as well as whether or not you obtained informed written or verbal consent. If consent was waived for your study, please include this information in your statement as well.

RESPONSE: Thank you for the comment. We have included a full ethics statement in the Methods section of the revised manuscript file including the full name of ethics committee that approved the study and waived the requirement of informed consent since data is irreversibly anonymized at the primary source (SISPRO).

5. We note that Figure 2 in your submission contain map images which may be copyrighted. All PLOS content is published under the Creative Commons Attribution License (CC BY 4.0), which means that the manuscript, images, and Supporting Information files will be freely available online, and any third party is permitted to access, download, copy, distribute, and use these materials in any way, even commercially, with proper attribution. For these reasons, we cannot publish previously copyrighted maps or satellite images created using proprietary data, such as Google software (Google Maps, Street View, and Earth). For more information, see our copyright guidelines: <http://journals.plos.org/plosone/s/licenses-and-copyright>.

We require you to either (1) present written permission from the copyright holder to publish these figures specifically under the CC BY 4.0 license, or (2) remove the figures from your submission:

a. You may seek permission from the original copyright holder of Figure 2 to publish the content specifically under the CC BY 4.0 license.

We recommend that you contact the original copyright holder with the Content Permission Form (<http://journals.plos.org/plosone/s/file?id=7c09/content-permission-form.pdf>) and the following text:

“I request permission for the open-access journal PLOS ONE to publish XXX under the Creative Commons Attribution License (CCAL) CC BY 4.0 (<http://creativecommons.org/licenses/by/4.0/>). Please be aware that this license allows unrestricted use and distribution, even commercially, by third parties. Please reply and provide explicit written permission to publish XXX under a CC BY license and complete the attached form.”

Please upload the completed Content Permission Form or other proof of granted permissions as an "Other" file with your submission.

In the figure caption of the copyrighted figure, please include the following text: “Reprinted from [ref] under a CC BY license, with permission from [name of publisher], original copyright [original copyright year].”

b. If you are unable to obtain permission from the original copyright holder to publish these figures under the CC BY 4.0 license or if the copyright holder’s requirements are incompatible with the CC BY 4.0 license, please either i) remove the figure or ii) supply a replacement figure that complies with the CC BY 4.0 license. Please check copyright information on all replacement figures and update the figure caption with source information. If applicable, please specify in the figure caption text when a figure is similar but not identical to the original image and is therefore for illustrative purposes only.

The following resources for replacing copyrighted map figures may be helpful:

USGS National Map Viewer (public domain): <http://viewer.nationalmap.gov/viewer/>

The Gateway to Astronaut Photography of Earth (public domain): <http://eol.jsc.nasa.gov/sseop/clickmap/>

Maps at the CIA (public domain): <https://www.cia.gov/library/publications/the-world-factbook/index.html> and <https://www.cia.gov/library/publications/cia-maps-publications/index.html>

NASA Earth Observatory (public domain): <http://earthobservatory.nasa.gov/>

Landsat: <http://landsat.visibleearth.nasa.gov/>

USGS EROS (Earth Resources Observatory and Science (EROS) Center) (public domain): <http://eros.usgs.gov/>#

Natural Earth (public domain): <http://www.naturalearthdata.com/>

RESPONSE: Thank you for the comment. The figures were elaborated directly by the authors and are not subject to copyright. We used the software QGIS (2009) which is an open access, open code geographical data system. The shapes/layers used to elaborate the map come from a public domain, open access and free use data available at <https://www.datos.gov.co/Mapas-Nacionales/Departamentos-y-municipios-de-Colombia/xdk5-pm3f>

This public domain is covered by Law 1712 of 2014 of transparency and access to national public information of Colombia which states that citizens (e.g., the authors) can access this public data freely and without restrictions and can be used by third parties. We have updated the figure caption of the copyrighted figure as follows: “Elaborated by the authors. Map shapes/layers from <https://www.datos.gov.co/Mapas-Nacionales/Departamentos-y-municipios-de-Colombia/xdk5-pm3f> under a CC BY license, covered by Law 1712 of 2014 of the Colombian Ministry of Information and Communication Technologies, 2022.”

Additional Editor Comments:

Dear Dr. Waich

We appreciate your submission to PLOS ONE. Both reviewers have provided a variety of important concerns and helpful suggestions. Please respond carefully to their suggestions. In particular, please pay attention to the critical point raised by Reviewer 2 as regards the diversity of methods used for the diagnosis of sleep apnea in your study.

Kuo-Cherh Huang


[Note: HTML markup is below. Please do not edit.]

Reviewers' comments:

Reviewer's Responses to Questions

**Comments to the Author**

**1. Is the manuscript technically sound, and do the data support the conclusions?**

**The manuscript must describe a technically sound piece of scientific research with data that supports the conclusions. Experiments must have been conducted rigorously, with appropriate controls, replication, and sample sizes. The conclusions must be drawn appropriately based on the data presented.**

Reviewer #1: Yes

Reviewer #2: No

2. Has the statistical analysis been performed appropriately and rigorously?

Reviewer #1: Yes

Reviewer #2: No

3. Have the authors made all data underlying the findings in their manuscript fully available?

The [PLOS Data policy](http://www.plosone.org/static/policies.action#sharing) requires authors to make all data underlying the findings described in their manuscript fully available without restriction, with rare exception (please refer to the Data Availability Statement in the manuscript PDF file). The data should be provided as part of the manuscript or its supporting information, or deposited to a public repository. For example, in addition to summary statistics, the data points behind means, medians and variance measures should be available. If there are restrictions on publicly sharing data—e.g. participant privacy or use of data from a third party—those must be specified.

Reviewer #1: Yes

Reviewer #2: Yes

4. Is the manuscript presented in an intelligible fashion and written in standard English?

PLOS ONE does not copyedit accepted manuscripts, so the language in submitted articles must be clear, correct, and unambiguous. Any typographical or grammatical errors should be corrected at revision, so please note any specific errors here.

Reviewer #1: Yes

Reviewer #2: Yes

5. Review Comments to the Author

Please use the space provided to explain your answers to the questions above. You may also include additional comments for the author, including concerns about dual publication, research ethics, or publication ethics. (Please upload your review as an attachment if it exceeds 20,000 characters)

Reviewer #1: Introduction
- line 57, Obstructive sleep-disordered breathing (oSDB) is a condition that encompasses breathing problems when asleep, due to an obstruction of the upper airways, ranging in severity from simple snoring to obstructive sleep apnoea syndrome (OSAS). It affects both children and adults. In children, hypertrophy of the tonsils and adenoid tissue is thought to be the commonest cause of oSDB. As such, tonsillectomy - with or without adenoidectomy - is considered an appropriate first-line treatment for most cases of paediatric oSDB. In otherwise healthy children, without a syndrome, of older age (five to nine years), and diagnosed with mild to moderate OSAS by PSG, there is moderate quality evidence that adenotonsillectomy provides benefit in terms of quality of life, symptoms and behaviour as rated by caregivers and high quality evidence that this procedure is beneficial in terms of PSG parameters. At the same time, high quality evidence indicates no benefit in terms of objective measures of attention and neurocognitive performance compared with watchful waiting. Furthermore, PSG recordings of almost half of the children managed non-surgically had normalised by seven months, indicating that physicians and parents should carefully weigh the benefits and risks of adenotonsillectomy against watchful waiting in these children. This is a condition that may recover spontaneously over time. For non-syndromic children classified as having oSDB on purely clinical grounds but with negative PSG recordings, the evidence on the effects of adenotonsillectomy is of very low quality and is inconclusive.Low-quality evidence suggests that adenotonsillectomy and CPAP may be equally effective in children with Down syndrome or MPS diagnosed with mild to moderate OSAS by PSG. please discuss and cite doi:10.1002/14651858.CD011165.pub2

RESPONSE: Thank you for the comment, we have added additional data in the revised manuscript regarding oSDB and the evidence of the effect of adenotonsillectomy and other management options in PSG values and other aspects of disease in children. We have discussed and cited the recommended reference.

- line 65, an interesting systematic review analyzed the correlation between changes in behavior and cognitive outcomes after AT were according to the scores post-AT in almost all studies. After comparing the AT group and control group, only one study had no difference that reached significance at one year post-AT. In another study, it did not show any significant improvement in terms of all behavioural and cognitive outcomes. The questionnaires on sleep-related quality of life after AT (PSQ-SRBD or ESS or OSA-18 or KOSA) may improve with positive changes in sleep parameters (AHI, ODI and SpO2). Furthermore, there is a significantly higher decrease in OSAS symptoms than the pre-AT baseline score. please discuss and cite doi:10.3390/children8100921

RESPONSE: Thank you for the comment, we have discussed and cited the recommended reference. Also, we added a sentence regarding the evidence of adenotonsillectomy and other therapeutical procedures to treat OSA improving PSG values, symptoms and quality of life of children with OSA.

Methods
Please apply the latest strobe guidelines, consort model and equator.

RESPONSE: Thank you for the comment. Since our study is observational, we have applied the latest STROBE guidelines.

Discussion
- To assess whether partial removal of the tonsils (intracapsular tonsillotomy) is as effective as total removal of the tonsils (extracapsular tonsillectomy) in relieving signs and symptoms of oSDB in children, and has lower postoperative morbidity and fewer complications. For children with oSDB selected for tonsil surgery, tonsillotomy probably results in a faster return to normal activity (four days) and in a slight reduction in postoperative complications requiring medical intervention in the first week after surgery. This should be balanced against the clinical effectiveness of one operation over the other. However, this is not possible to determine in this review as data on the long-term effects of the two operations on oSDB symptoms, quality of life, oSDB recurrence and need for reoperation are limited and the evidence is of very low quality leading to a high degree of uncertainty about the results. please discuss and cite doi:10.1002/14651858.CD011365.pub2

RESPONSE: Thank you for the comment, we have discussed and cited the recommended reference. We added a sentence regarding the different therapeutic options existing for pediatric SA and that some have variations such as intracapsular vs. extracapsular adenotonsillectomy since these specific data does not tend to appear in health registries such as SISPRO.

Reviewer #2: Dear author,
I appreciate the fact that your article first focused on the analysis of the prevalence in the pediatric population of sleep apnea in Colombia. It is definitely a good starting point for the study of this pathology now widely spread and of great interest to the general population. It is evident, when reading the article, the difficulties encountered in data collection and the diversity of methods used for the diagnosis of sleep apnea.

My advice is to prefer standard diagnostic tools for apnea such as polysomnography or pulse oximetry (preferred method in pediatric age). This would also allow a better differential diagnosis between central and obstructive apneas. You could try to conduct a nationwide multicenter study rather than the Colombian registry analysis to circumvent this problem. Therefore, you could use the data you collected on posing about the lack of standard methods for the study of apnea and not just subdiagnosis. I wish you a good continuation of your work.

RESPONSE: Thank you for the comment and review. We added new sentences throughout the revised manuscript regarding the diversity of diagnostic methods for SA. Since the patients in our study were given a **confirmed** principal diagnosis of SA by a healthcare professional, they should have undergone polysomnographic testing confirming SA (central and/or obstructive).

We agree with the reviewer on the limitations that are inherent to the use of a healthcare registries methodology to collect data and that are reflected in the discussion section of the manuscript. We agree with the reviewer in that a nationwide multicenter study could allow for a better differential diagnosis between central and obstructive apneas, our study would also invite healthcare authorities to start discriminating between central and obstructive sleep apneas in the national health registry. We expect this first analysis to invite researchers to design and perform studies with different methodologies such as a nationwide multicenter study proposed by the reviewer, we have added further insight in this matter in the discussion section of the revised manuscript.

In the future, a prospective study is proposed to include patients diagnosed with sleep apnea using PSG as a diagnostic method to achieve greater precision between central and obstructive sleep apnea.

6. PLOS authors have the option to publish the peer review history of their article ([what does this mean?](https://journals.plos.org/plosone/s/editorial-and-peer-review-process#loc-peer-review-history)). If published, this will include your full peer review and any attached files.


If you choose “no”, your identity will remain anonymous but your review may still be made public.


Do you want your identity to be public for this peer review? For information about this choice, including consent withdrawal, please see our [Privacy Policy](https://www.plos.org/privacy-policy).

Reviewer #1: No

Reviewer #2: No

[NOTE: If reviewer comments were submitted as an attachment file, they will be attached to this email and accessible via the submission site. Please log into your account, locate the manuscript record, and check for the action link "View Attachments". If this link does not appear, there are no attachment files.]

While revising your submission, please upload your figure files to the Preflight Analysis and Conversion Engine (PACE) digital diagnostic tool, <https://pacev2.apexcovantage.com/>. PACE helps ensure that figures meet PLOS requirements. To use PACE, you must first register as a user. Registration is free. Then, login and navigate to the UPLOAD tab, where you will find detailed instructions on how to use the tool. If you encounter any issues or have any questions when using PACE, please email PLOS at [figures@plos.org](mailto:figures@plos.org). Please note that Supporting Information files do not need this step.

RESPONSE: Thank you for the comment, we have uploaded the updated figure files to PACE digital diagnostic tool.
